# Neighborhood Socioeconomic Disadvantage and Frailty among Mid-to-Older-Aged Adults in Australia: Cross-Sectional and Longitudinal Associations

**DOI:** 10.1007/s11524-025-01018-2

**Published:** 2025-10-20

**Authors:** Takumi Abe, Takemi Sugiyama, Alison Carver, Manoj Chandrabose, Gavin Turrell

**Affiliations:** 1 School of Commerce, Meiji University, Tokyo, Japan; 2 School of Health Science, Swinburne University of Technology, Melbourne, VIC Australia; 3 School of Social Sciences, Media, Film and Education, Swinburne University of Technology, Melbourne, VIC Australia; 4 National Centre for Healthy Ageing, Frankston, VIC Australia; 5 School of Translational Medicine, Monash University, Melbourne, VIC Australia; 6 Centre for Urban Research, RMIT, Melbourne, VIC Australia

**Keywords:** Deprivation, Inequalities, Neighborhood environment, Physical function

## Abstract

This study examined the cross-sectional and longitudinal associations between neighborhood socioeconomic disadvantage and frailty in middle-to-older-aged adults. Data were obtained from a cohort study conducted in Brisbane, Australia, with 3966 participants (mean age 58.2 years; female 57.6%) included in cross-sectional analysis and 2846 in longitudinal analysis (3-year follow-up). A frailty index (FI), a composite score consisting of 32 items, was used continuously (range 0–1) and categorized into frail (FI > 0.35) and non-frail. Neighborhood disadvantage was measured using the Index of Relative Socioeconomic Disadvantage, an area-level composite index, and was categorized into tertiles. Two-level mixed-effects linear and logistic regression models were fitted, accounting for area-level clustering through random intercepts for areas and adjusting for demographic variables and individual-level socioeconomic status. In the cross-sectional analysis, 11% of participants were frail. Compared to residents of low disadvantage neighborhoods, those in middle and high disadvantage neighborhoods had higher levels of frailty and greater odds of being frail (OR = 1.54 [95% CI 1.13, 2.09] and OR = 2.86 [2.11, 3.88], respectively). In the longitudinal analysis, residing in middle and high disadvantage neighborhoods was significantly associated with increases in FI scores, relative to low disadvantage neighborhoods. Incident frailty was observed in 5% of participants at the follow-up. Residing in high disadvantage neighborhoods showed significantly higher odds of incident frailty (OR = 1.75 [1.08, 2.86]). Mid-to-older-aged residents of disadvantaged neighborhoods are more likely to be frail and to become frail over 3 years, compared to those in advantaged neighborhoods. Community-level interventions to reduce frailty should target disadvantage neighborhoods.

**Supplementary Information:**

The online version contains supplementary material available at 10.1007/s11524-025-01018-2.

 Frailty, characterized by multidimensional (i.e., physical, mental, and cognitive) functional decline, leading to increased vulnerability to stressors, is known to elevate the risk of adverse health outcomes, such as hospitalization, institutionalization, dementia, and mortality [1–3]. It also has a significant impact on public health systems, as more resources (care facilities and personnel) are needed to look after those with frailty. Frailty is an emerging public health issue in many countries, mainly due to the ageing population. In particular, in Australia, “baby boomers,” a large population subgroup born between 1946 and 1964, are now in their 60s and 70s. It has been shown that 11% of Australian adults 65 years and over were frail in 2016, and this is expected to increase to 16% in 2027 [4]. Reducing frailty is a key target for public health due to its significant social cost and increasing trend [4, 5]. The prevalence of frailty increases with age; however, this does not imply that frailty is a health concern only among older adults. A meta-analysis of population-based studies from 62 countries identified that 23% of individuals who are in their 50s are frail [6], based on deficit accumulation models that predict likelihood of frailty using a count of physical, mental, and cognitive disorders and impairments [7]. A UK Biobank study found increased mortality risk in both frail middle-aged and older adults [8]. Hence, preventing frailty is important not only for older adults but also for middle-aged adults. Frailty is not evenly distributed across society [9]. Systematic reviews found consistent associations between frailty and individual-level measures of socio-economic status (SES), such as education and income: those with lower SES are more likely to be frail compared to those with higher SES [10, 11]. However, in contrast, the reviews found just a few studies investigating area-level socio-economic disadvantage as an exposure. Area-level investigation is useful because where people live may affect their ageing process by shaping their daily health-related behaviors (e.g., physical activity, sedentary behavior and social interaction with others), which can affect their long-term health independent of their individual characteristics [12, 13]. This reasoning—that neighborhood environments may influence health outcomes through those behaviors—also applies to frailty research. For instance, a previous study found that the association between neighborhood environments and frailty is partially mediated by interpersonal and intrapersonal factors, such as physical activity and social support [14]. Because frailty is associated with a wide range of factors [15], the specific pathways leading to frailty likely differ between individuals. Therefore, focusing on environmental factors, given their potential to influence multiple individual-level factors [16], may help provide a broader perspective on the multifaceted nature of frailty, rather than a detailed understanding of any single pathway. A cross-sectional association between living in disadvantaged neighborhoods and a higher level of frailty has been found in the Netherlands [17] and England [18]. A longitudinal study in England found that neighborhood deprivation was associated with a greater level of frailty incidence among middle-aged and older adults [19]. Within-country distribution of frailty according to neighborhood disadvantage has been examined mainly in European countries, with little investigation in other countries. This is an important research gap that needs to be addressed, given that studies have demonstrated between-region differences in the prevalence of frailty [6, 20]. Investigation of whether neighborhood disadvantage is associated with frailty, among both middle-aged and older adults, in non-European countries can inform local public health policies seeking to reduce frailty and its unequal distribution (if any). This study examined the cross-sectional and longitudinal associations between neighborhood disadvantage and frailty in middle-to-older-aged adults in Brisbane, Australia.

## Methods

### Data Source and Study Participants

We used data from the How Areas in Brisbane Influence HealTh and AcTivity (HABITAT) study, a longitudinal cohort. The primary aim of HABITAT was to study the prevalence and incidence of health outcomes, and their determinants, among mid-to-older-aged adults. The details of the sampling design and recruitment procedures have been described elsewhere [21, 22]. Briefly, at baseline in 2007, a multi-stage probabilistic sampling design was used to select study areas and participants. Study areas were defined as Census Collection Districts (CCDs), the smallest geographical units used by the Australian Bureau of Statistics for census data collection at the time of the baseline HABITAT study [23]. Each CCD contained, on average, approximately 200 occupied private dwellings. From the 1680 CCDs in Brisbane, a stratified random sample of 200 CCDs was selected. In each selected CCD, an average of 85 households was selected, and from each household, a random sample of individuals aged 40–65 years was chosen to participate [21]. The HABITAT baseline survey was conducted in 2007 when 17,000 self-administered questionnaires were sent by mail. Of 16,128 respondents, 11,035 were deemed eligible (response rate of 68.4%; Supplementary Table 1). They were found to reflect the sociodemographic characteristics of the Brisbane population [22]. Follow-up surveys were conducted at four times: Wave 2 (W2) in 2009, Wave 3 (W3) in 2011, Wave 4 (W4) in 2013, and Wave 5 (W5) in 2016.

We used data collected in W4 (*n* = 6520) and W5 (*n* = 5187), as sufficient health-related questions to calculate a frailty index (FI) were asked only in these two most recent waves. We considered participants eligible for the cross-sectional analysis as those who did not move from the baseline to W4 to ensure that they lived in the same CCD (exposed to the same socio-economic condition) for at least 6 years. A total of 4339 participants were eligible. The HABITAT study was approved by the University Human Research Ethics Committee at the Queensland University of Technology (ID3967H).

### Measures

#### Frailty Assessment

We previously developed a FI using the HABITAT data [24] according to the standardized procedure established previously [25]. The FI was composed of 32 items, covering medical history, signs and symptoms of health and geriatric conditions, mental health, and physical function and activity (Supplementary Table 2). The FI was calculated by dividing the total raw score by the number of items and ranges from 0 to 1, with a higher score indicating a greater level of frailty. Continuous FI scores were used to assess the level of frailty, while a threshold of > 0.35 was applied to categorize participants into frail and non-frail [26–29].

#### Neighborhood Socioeconomic Disadvantage

We used the Index of Relative Socioeconomic Disadvantage (IRSD), an area-level composite index developed by the Australian Bureau of Statistics, with 17 area-level disadvantage measures such as the proportion of people with low income, lower education attainment, and unemployment [30, 31]. The IRSD values of the participants’ neighborhoods (CCDs) at W4 were used as the exposure in our analyses. Those values were first transformed into percentiles relative to the whole of Brisbane and then categorized into tertiles to represent high disadvantage neighborhoods (most disadvantaged, IRSD percentile ≤ 33%), middle disadvantage neighborhoods (moderately disadvantaged, IRSD percentile 34–66%), and low disadvantage neighborhoods (least disadvantaged, IRSD percentile ≥ 67%).

### Data Analysis

**Fig. 1:**
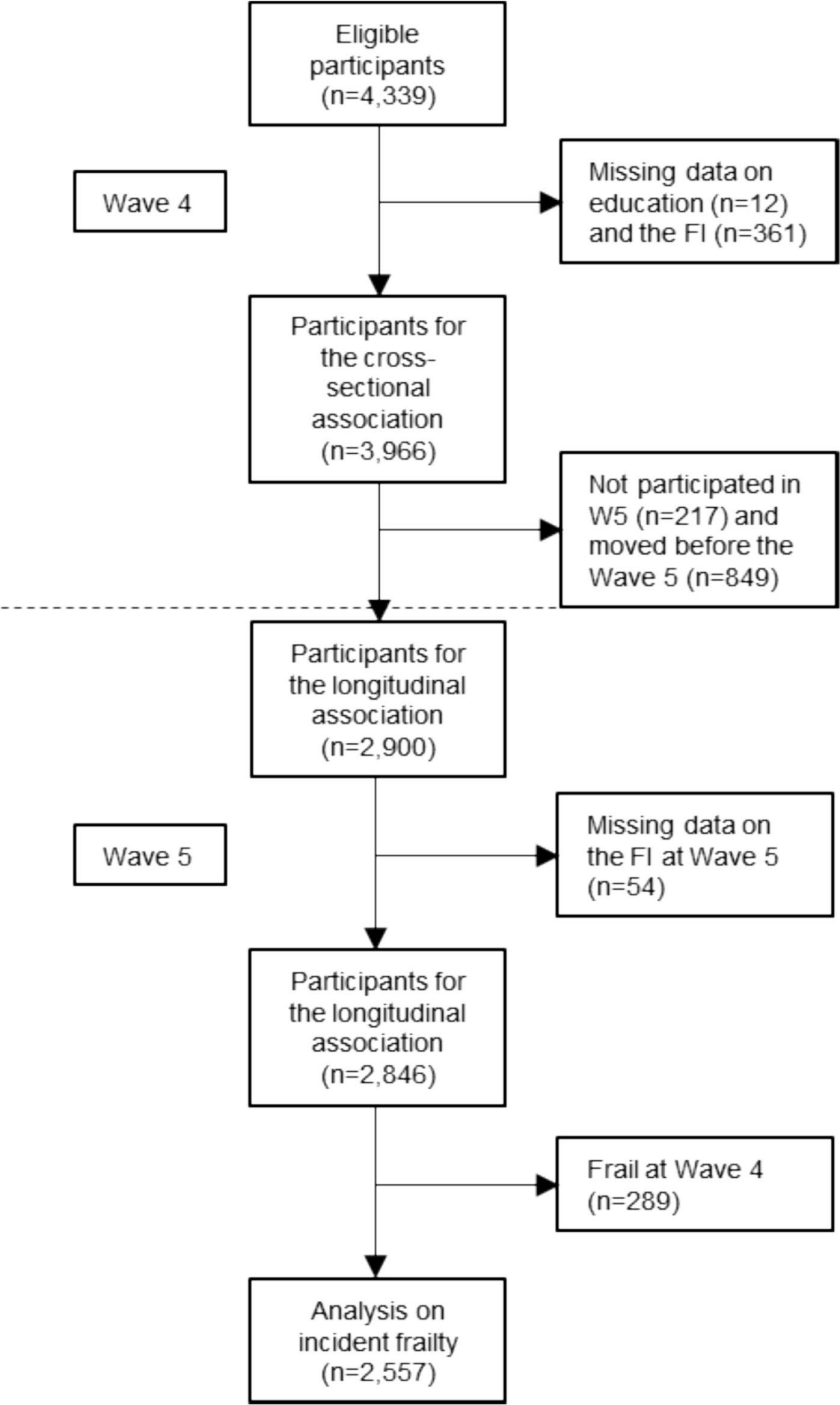
Eligible participants were those who did not change their address between Waves 1 (in 2007) to 5 (in 2016). For the cross-sectional analysis, 11% (*n* = 424) of participants were frail. For the longitudinal analysis, 5% (*n* = 125) of participants became frail

Figure 1 shows a flowchart of how analytical samples were derived. A “missing” category was created for covariates, except for education, where only 12 participants had incomplete data. After removing participants who had missing data for education and for over 20% of the FI items (7 items and more) [32], the cross-sectional analysis included 3966 participants. For the longitudinal analysis, we further excluded those who moved to another address between W4 and W5 and did not meet the criteria to calculate FI. This left 2846 participants. Among those, participants who were non-frail at W4 were eligible for examining the incidence of frailty (*n* = 2557). All analyses were conducted using Stata 17 (StataCorp, College Station, TX, USA). Statistical significance was set at 0.05.

### Cross-Sectional Analysis

To examine the cross-sectional associations at W4, we used the level of frailty (i.e., continuous FI scores) and the status of frailty (i.e., a binary measure of being frail or not) as outcome measures. The exposure was the level of neighborhood disadvantage described above. We fitted two-level (i.e., participants at level 1 and CCDs at level 2) mixed-effect linear and logistic regression models to assess the relevant associations. The models accounted for area-level clustering by including random intercepts for CCDs [33]. Two models were fitted: Model 1 adjusted for age and sex and Model 2 further adjusted for living arrangement, education, working status, and household income.

### Longitudinal Analysis

For longitudinal analyses, we examined associations of area-level socioeconomic disadvantage at W4 with the individual-level change in the level of frailty between W4 and W5 (i.e., a continuous measure of change) and the incidence of frailty at W5 among those who were non-frail at W4 (i.e., a binary measure of incidence). As described above, we employed two-level mixed-effect linear and logistic regression models to assess the associations. Two models with a different set of covariates mentioned above (Models 1 and 2) were fitted. Additionally, a paired *t*-test was used to compare FI between W4 and W5.

## Results

### Characteristics of the Study Samples

Table 1 shows the characteristics of the participants included in cross-sectional and longitudinal analyses, as well as the baseline participants of the HABITAT study. About three-quarters of the participants were middle-aged people aged < 65 years old in the cross-sectional and longitudinal samples. The proportions of participants with higher levels of education and income, living alone, and not employed were greater in our analytical samples compared to the baseline. Characteristics of participants by neighborhood disadvantage tertiles are shown in Supplementary Table 3.

**Table 1:** Characteristics of participants for cross-sectional and longitudinal analyses

	Participants for the cross-sectional analysis (*n* = 3966)	Participants for the longitudinal analysis (*n* = 2846)
% < 65 years at W4	76.0	76.6
Sex, % women	57.6	57.8
Education, %		
Bachelor’s degree or higher	35.2	37.6
Diploma/associate diploma	11.4	11.2
Certificate (trade/business)	17.1	17.6
School	36.3	33.5
Living arrangement at W4, %		
Living alone	16.5	16.0
Living with others	79.0	79.7
Other	2.9	2.7
Missing	1.6	1.6
Work status at W4, %		
Working	59.2	60.4
Not working	36.9	36.3
Missing	3.9	3.2
Household income at W4, %		
$130,000–	20.0	20.1
$72,800–$129,999	23.5	23.3
$52,000–$72,799	12.7	12.7
$26,000–$51,999	19.1	19.1
$0–$25,999	10.1	10.5
No answer to the question	14.7	14.4

### Descriptive Analyses

Descriptive results on frailty according to neighborhood disadvantage tertiles are presented in Table 2. In both analytical samples, about 46% of participants were in low disadvantage neighborhoods, 32% in middle disadvantage neighborhoods, and 22% in high disadvantage neighborhoods. The mean FI score of the cross-sectional sample was 0.22 (SD = 0.11), with 11% of them being frail. While the increase in mean FI score from W4 to W5 was statistically significant, the magnitude of this change was small (Cohen’s *d* = 0.18). The FI score at W5 was significantly higher than those at W4 (effect size: d = 0.18). Five percent of participants who were not frail at W4 became frail at W5. At both waves, there was a clear socioeconomic gradient in frailty; for instance, the prevalence of frailty at W4 ranged from 6% in low disadvantage neighborhoods to 20% in high disadvantage neighborhoods. Supplementary Table 4 shows descriptive data of area-level SES percentile and FI score for those who were included and those who were excluded (did not participate at W5, moved between W4 and W5, or incomplete FI score at W5) in the longitudinal analysis. Those who did not participate at W5 had a significantly lower mean IRSD percentile, a lower mean FI score at W4, and a higher prevalence of frailty at W4 compared to both the analytical sample for longitudinal analyses and those who moved between W4 and W5.

**Table 2:** Descriptive characteristics of frailty according to neighborhood disadvantage

	Neighborhood disadvantage level at W4			Total
	Low	Middle	High	
Cross-sectional analysis at W4				
* N*	1817 (45.8%)	1259 (31.7%)	890 (22.5%)	3966
Mean FI score at W4 (SD)	0.19 (0.09)	0.22 (0.11)	0.25 (0.13)	0.22 (0.11)
Frailty at W4, %	6.1	10.8	20.1	10.7
Longitudinal analysis from W4 to W5				
* N*	1318 (46.3%)	902 (31.7%)	626 (22.0%)	2846
Mean FI score at W4 (SD)	0.19 (0.09)	0.22 (0.11)	0.25 (0.13)	0.21 (0.11)
Mean FI score at W5 (SD)	0.21 (0.08)	0.23 (0.10)	0.26 (0.11)	0.22 (0.10)
Mean change in FI score from W4 to W5 (SD)	0.01 (0.06)	0.01 (0.07)	0.01 (0.07)	0.01 (0.07)
Frailty at W5, %	7.0	10.9	20.1	11.1
Incidence of frailty at W5, %†	3.6	5.2	7.5	4.9

†Among those who were not frail at W4 (*n* = 2557)

Frailty was defined as > 0.35 of the FI score

### Cross-Sectional Analyses

Compared to low disadvantage neighborhoods, residing in middle and high disadvantage neighborhoods was significantly associated with 0.03 and 0.06 greater FI scores, respectively (cross-sectional analysis Model 1, Table 3). These regression coefficients were attenuated slightly after further adjustment for individual-level SES but remained significant (Model 2). Relative to residing in low disadvantage neighborhoods, the odds of being frail were 1.9 times higher in middle disadvantage neighborhoods and 3.9 times higher in high disadvantage neighborhoods (Model 1). These associations were also attenuated but remained significant after adjusting for individual-level SES.

**Table 3:** Cross-sectional and longitudinal associations between neighborhood disadvantage and frailty

	Model	Neighborhood disadvantage level		
		Low	Middle	High
Cross-sectional analysis at W4				
Regression coefficient for FI score (95%CI)	1	Ref	0.03 (0.02, 0.04)***	0.06 (0.05, 0.07)***
	2	Ref	0.01 (0.006, 0.02)**	0.04 (0.03, 0.05)***
Odds ratio of being frail (95% CI)	1	Ref	1.89 (1.38, 2.57)***	3.91 (2.88, 5.29)***
	2	Ref	1.54 (1.13, 2.09)**	2.86 (2.11, 3.88)***
Longitudinal analysis from W4 to W5				
Regression coefficient for change in FI score (95%CI) ‡	1	Ref	0.02 (0.01, 0.03)***	0.04 (0.03, 0.06)***
	2	Ref	0.01 (0.01, 0.02)***	0.04 (0.03, 0.05)***
Odds ratio of becoming frail§ (95%CI) †	1	Ref	1.31 (0.81, 2.13)	2.11 (1.31, 3.40)**
	2	Ref	1.16 (0.71, 1.87)	1.75 (1.08, 2.86)*

*p <.05, **p <.01, ***p <.001

†Among those who were not frail at W4 (*n* = 2557)

‡Unchangeable variables (sex and education) were not included in the model

Frailty was defined as > 0.35 of the FI score

Model 1 adjusted for gender and age. Model 2 further adjusted for living alone, education, working status, and income. Both models accounting for clustering at the CCD level

### Longitudinal Analyses

Compared to residing in low disadvantage neighborhoods, residing in middle and high disadvantage neighborhoods was significantly associated with a 0.02 and 0.04 increase in FI scores, respectively, between W4 and W5 (longitudinal analysis Model 1, Table 3). These associations remained significant after further adjustments. Among those without frailty at W4, those residing in high disadvantage neighborhoods had 2.1 times higher odds of incident frailty compared to those residing in low disadvantage neighborhoods, which remained significant after further adjustment. There was no statistically significant difference in the odds of incident frailty between middle and low disadvantage neighborhoods.

## Discussion

Understanding how frailty among middle-to-older-aged adults is distributed within society is important for developing area-based public health strategies to prevent frailty. This study examined cross-sectional and longitudinal associations between neighborhood disadvantage and frailty in the context of Australia. We found significant area-level socioeconomic inequalities in frailty. Specifically, cross-sectional analyses showed that individuals residing in high disadvantage neighborhoods had higher levels of frailty compared to those in low disadvantage neighborhoods. Longitudinal analyses revealed that continued exposure to neighborhood disadvantage over time was linked to both increased frailty and the development of frailty. These findings point to the importance of targeting disadvantaged neighborhoods in public health initiatives to address frailty in ageing populations and its unequal distribution.

Previous studies conducted in European countries found that higher neighborhood socioeconomic disadvantage is associated with higher levels of frailty among middle-to-older-aged adults [17, 18]. Our results from the cross-sectional analysis support this finding and suggest that the cross-sectional association between neighborhood disadvantages and frailty is observed across various regions. Residents of disadvantaged neighborhoods tend to face difficulties in accessing healthcare [34], which can result in poor management of chronic diseases and worse health conditions [35]. Indeed, a study analyzing household-based panel data reported that healthcare use was more frequent among those with higher socioeconomic status than among those with lower in Australia [36]. Additionally, residents of disadvantaged neighborhoods are prone to have unhealthy lifestyle [37]. While few studies have examined mediating factors linking neighborhood environment and frailty, some factors, such as the number of diseases and low levels of physical activity, have been reported as potential mediators. The accumulation of such negative impacts of living in disadvantaged neighborhoods, which could exacerbate these factors, may lead to health deficits, as assessed using the FI.

We found that 5% of non-frail middle-to-older-aged adults became frailty in 3 years. A meta-analysis that calculated incident frailty in non-frail adults aged ≥ 60 years reported that it was 43.4/1000 person-year [38], while incident frailty in this study was equivalent to 16.3/1000 person-year. Given that approximately three-quarters of the study sample were adults aged ≤ 64 years, it would be reasonable that the incidence rate we observed was lower than the meta-analysis.

Our findings suggest an adverse impact of exposure to neighborhood disadvantage on frailty levels and on becoming frail. Those excluded from the longitudinal analysis, particularly due to no participation at W5 and incomplete FI at W5, had higher FI scores at W4 than those who were included. This suggests that those with lower frailty levels tended to remain in the longitudinal analysis. Despite this, we found the longitudinal association between neighborhood disadvantage and frailty to be statistically significant. Previous studies showed inconsistent results regarding the association between neighborhood disadvantage and change in frailty levels. For example, a Chinese study did not find a significant association between community-level SES and frailty levels [39], while a study from England reported that living in a deprived area increases the risk of becoming frail [19]. Our study supported the latter finding. It is possible that the way frailty is distributed according to neighborhood disadvantage may differ between different cultural contexts. The difference in frailty levels between high and low individual-level SES would diminish with advancing age. This may not be apparent in middle and early old age but becomes more noticeable in later old age [40]. This trend may apply to area-level SES disadvantage. Indeed, in our analytical sample, approximately three-quarters of the analytical sample were middle-aged participants, similar to the England study, where approximately half of the participants were in their 50s. In contrast, the Chinese study focused on individuals aged ≥ 60 years, with a mean age of 68 years. Although we adjusted for age in examining the association between neighborhood disadvantage and frailty, the accumulated influence on health status of exposure to neighborhood disadvantage for a long time may not be eliminated. These findings indicate that the age composition of the study sample may influence the association between neighborhood disadvantage and frailty. Future research may investigate the age-specific distribution of frailty (e.g., middle-aged, 65–74 years, 75 + years). A British cohort study suggests that interventions that target healthy behaviors (e.g., physical activity, smoking cessation) and management of cardiometabolic risk factors in middle age may help reduce individual-level socioeconomic differences in frailty later in life [41]. As with individual-level SES, further research is needed to identify factors that mitigate the negative impact of residing in disadvantaged neighborhoods on frailty.

The FI was treated as a continuous outcome variable in this and previous studies [17, 18], which can highlight differences in frailty levels across neighborhood disadvantage levels. We also used the FI to create a binary outcome variable indicating frailty status (i.e., frail or not) and found that middle-to-older-aged adults identified as frail are more prevalent in highly disadvantaged neighborhoods than in less disadvantaged neighborhoods. This finding is important when developing approaches to address frailty, as interventions should be tailored according to the degree of frailty [42]. Although further evidence is needed regarding effective strategies at the community level [43], community-based interventions may help prevent the progression to conditions requiring daily support or care [44]. To implement such interventions effectively, municipalities and public health authorities need to identify localities with a high prevalence of frailty. In this context, neighborhood disadvantage may serve as a practical indicator for prioritizing resource allocation.

An advantage of focusing on neighborhood disadvantage is that it enables the identification of priority areas for frailty prevention and care. This perspective facilitates the efficient allocation of resources to address frailty within limited capacity. Another advantage is that neighborhood disadvantage can be assessed using public resources, simplifying the assessment process. Given these advantages, our findings are particularly useful for public health sectors and practitioners in community settings. Nevertheless, the generalizability of our findings requires careful interpretation. We used data from W4 and W5 of HABITAT, which have slightly different characteristics (e.g., participants with higher educational attainment were retained), compared to baseline data that were found to be representative of the Brisbane population (Supplementary Table 1) [22]. Additionally, the assessment of neighborhood disadvantage was based on the IRSD, which is tailored to the Australian context. Therefore, further research is needed to explore the applicability of our findings in other regions within and beyond Australia. Another limitation is that some bias may arise. Self-reporting bias, which occurs frequently due to the nature of the FI (i.e., being composed of self-reported items), would be inevitable in this study. Although we developed the FI according to the standardized procedure established previously [25], the bias may have led to lower FI scores, especially when participants insufficiently grasp their health and functional status. Selection bias, especially attrition bias, is another concern, as 28% of participants included in the cross-sectional analyses were excluded from the longitudinal analyses, mainly due to non-participation at W5. Even though this attrition may have biased the association toward the null due to the characteristics of those who did not participate in the survey at W5 (tendency of residing in disadvantaged areas and having higher FI scores), we still found the significant association between neighborhood disadvantage and frailty. We acknowledged that the follow-up period of 3 years in this study was shorter than that in previous studies [19, 39]. Even though the short follow-up period resulted in a small mean change in the FI score from W4 to W5, the study found distinct patterns of longitudinal findings for different levels of neighborhood disadvantage. It can be argued that our study supports the relevance of neighborhood disadvantage to residents’ frailty and its trajectory.

## Conclusions

In conclusion, we found that neighborhood socioeconomic disadvantage was a factor related to residents’ frailty status and development of frailty over time, in a large sample of middle-to-older-aged adults. Our findings suggest that, even after accounting for individual-level SES, those who reside in high disadvantage areas are more likely than those in low disadvantage areas to be frail and to become frailty over time. Future research needs to identify underlying social and environmental specific factors that contribute to a greater level of frailty in disadvantaged neighborhoods. Interventions need to be developed based on findings from such research to reduce the prevalence and incidence of frailty in disadvantaged neighborhoods.

## Supplementary Information

Below is the link to the electronic supplementary material.

ESM1 (DOCX 133 KB)

## Data Availability

This study used data from HABITAT, the availability of which is described in a cohort profile paper [16].
