# Supplementary material for: Neighborhood Socioeconomic Disadvantage and Frailty among Mid-to-Older-Aged Adults in Australia: Cross-Sectional and Longitudinal Associations
Source: J Urban Health. 2025 Oct 20;102(5):1119–29. doi: 10.1007/s11524-025-01018-2 (PMC12669419; doi:10.1007/s11524-025-01018-2)
Supplement: Supplementary file 1 — (DOCX 133 KB) [file 11524_2025_1018_MOESM1_ESM.docx]

Supplementary Table 1. Characteristics of initial participants of the HABITAT cohort

|  | HABITAT cohort at baseline (n=11,035) |
| --- | --- |
| % ≥65 years | 0 |
| Sex, % women | 56.1 |
| Education, % |  |
| Bachelor's degree or higher | 31.3 |
| Diploma/associate diploma | 11.5 |
| Certificate (trade/business) | 17.7 |
| School | 39.1 |
| Living arrangement, % |  |
| Living alone | 14.6 |
| Living with others | 84.1 |
| Other | 0.3 |
| Missing | 1.0 |
| Work status, % |  |
| Working | 76.1 |
| Not working | 19.4 |
| Missing | 4.5 |
| Household income, % |  |
| $130,000– | 17.1 |
| $72,800–$129,999 | 25.8 |
| $52,000–$72,799 | 14.7 |
| $26,000–$51,999 | 18.2 |
| $0–$25,999 | 9.5 |
| No answer to the question | 14.7 |

Data on education includes 0.4% of missing, and that on work status had 6.3% of ‘other category’, which means types of works that cannot be categorised using the Australian and New Zealand Standard Classification of Occupations.

Supplementary Table 2. Components of the frailty index

| **Medical history (n=8)** | | **Response format** |
| --- | --- | --- |
|  | Chronic bronchitis or emphysema | Yes =1; No =0 |
|  | Cancer |  |
|  | Diabetes |  |
|  | Heart/coronary disease |  |
|  | Osteoporosis |  |
|  | Arthritis |  |
|  | Asthma |  |
|  | Serious circulatory condition (e.g. stroke, hardening of the arteries) |  |
| **Signs and symptoms (n=9)** | |  |
|  | General health | Poor/fair =1; Good =0.5; Very good/excellent =0 |
|  | Sleep quality (during the last week) |  |
|  | Back pain (in the last 12 months) | Often =1; Sometimes =0.67; Rarely =0.33; Never =0 |
|  | Severe tiredness (in the last 12 months) |  |
|  | Breathing difficulties (in the last 12 months) |  |
|  | Isolated from others |  |
|  | Eyesight problems |  |
|  | Activities of daily living support | Yes =1; No =0 |
|  | Body mass index (kg/m^2^) | <18.5 or ≥30 =1; 18.5–29.9 =0 |
| **Mental health (n=8)** | |  |
|  | Satisfied with your life | 1–3 pts =1; 4–7 pts =0.5; 8–10 pts =0 |
|  | Nervous (in the last four week) | All of the time/most of the time =1; Some of the time =0.5; None/a little of the time =0 |
|  | Hopeless (in the last four weeks) |  |
|  | Sad (in the last four weeks) |  |
|  | Worthless (in the last four weeks) |  |
|  | Lots of energy (in the last four weeks) |  |
|  | Feeling relaxed (in the last two weeks) | None of the time/rarely =1; Some of the time =0.5; Often/all of the time =0 |
|  | Thinking clearly (in the last two weeks) |  |
| **Physical function and activity (n=7)** | |  |
|  | Lifting/carrying groceries | Yes, limited a lot =1; Yes, limited a little =0.5; No, not limited at all =0 |
|  | Climbing one flight of stairs |  |
|  | Bending, kneeling, and stooping |  |
|  | Walking more than 1 km |  |
|  | Bathing or dressing yourself |  |
|  | Health restricted you from PA |  |
|  | Engaging in physical activity (continuous walking at least 10 minutes in the last week) | None =1; One or more =0 |

Supplementary Table 3. Characteristics of participants according to neighbourhood disadvantage

|  | Neighbourhood disadvantage level | | |
| --- | --- | --- | --- |
|  | Low  (n=1,817) | Medium  (n=1,259) | High  (n=890) |
| Age group, % ≥65 years | 21.2 | 25.1 | 28.2 |
| Sex, % women | 57.2 | 56.6 | 59.9 |
| Education, % |  |  |  |
| Bachelor's degree or higher | 44.8 | 30.6 | 22.9 |
| Diploma/associate diploma | 12.7 | 9.9 | 10.8 |
| Certificate (trade/business) | 14.1 | 20.3 | 18.8 |
| School | 28.7 | 39.2 | 47.5 |
| Living arrangement |  |  |  |
| Living alone | 11.6 | 19.9 | 21.6 |
| Living with others^†^ | 84.7 | 76.9 | 71.5 |
| Other | 2.3 | 2.7 | 4.5 |
| Missing | 1.5 | 1.3 | 2.5 |
| Work status |  |  |  |
| Working | 63.5 | 58.3 | 51.8 |
| Not working | 32.8 | 37.6 | 44.5 |
| Missing | 3.8 | 4.1 | 3.7 |
| Household income |  |  |  |
| $130,000– | 29.3 | 14.3 | 9.1 |
| $72,800–$129,999 | 24.6 | 24.5 | 19.9 |
| $41,600–$72,799 | 16.4 | 23.4 | 23.2 |
| $0–$41,599 | 15.0 | 24.1 | 31.8 |
| Missing | 14.8 | 13.7 | 16.1 |

|  | **Included** | **Excluded** | | | ***p*** | **Post-hoc analysis** |
| --- | --- | --- | --- | --- | --- | --- |
|  | (n=2,846) ^a^ | Not participate at W5  (n=849) ^b^ | Moved between W4 & W5 (n=217) c | Incomplete FI score at W5 (n=54) ^d^ |  |  |
| Mean IRSD percentile (SD) | 57.8 (27.3) | 54.7 (27.1) | 61.8 (25.8) | 51.5 (29.3) | <.001 | b < a, c |
| Mean FI score at W4 (SD) | 0.21 (0.11) | 0.22 (0.11) | 0.20 (0.09) | 0.25 (0.11) | <.001 | a, c < b, d |
| Frailty at W4, % | 10.2 | 13.6 | 6.0 | 14.8 | .003 | a, c < b |

Supplementary Table 4. Comparisons of area-level SES and frailty between included and excluded participants in the longitudinal analyses

IRSD: Index of Relative Socioeconomic Disadvantage

P values were obtained from ANOVA for continuous outcomes shown as mean (SD) and χ^2^ test for proportional outcome in the table.

Post hoc tests were conducted using a Bonferroni correction.

Note. Frailty was defined as >0.35 of the FI score.
